# Management of Patients With Post-Traumatic Wrist Flexor Contracture by Carpectomy and Wrist Fusion

**DOI:** 10.7759/cureus.48812

**Published:** 2023-11-14

**Authors:** Sriman Narayan Madan Mohan, Subashini Rajendiran, Srinivasan Rajappa

**Affiliations:** 1 Department of Orthopaedics, Sri Ramachandra Institute of Higher Education and Research, Chennai, IND; 2 Department of Hand Surgery, Sri Ramachandra Institute of Higher Education and Research, Chennai, IND

**Keywords:** reconstruction, wrist fusion, wrist flexor contracture, trauma, carpectomy

## Abstract

Post-traumatic wrist flexor contracture is most commonly seen in major trauma affecting the hand, wrist, and forearm. It produces debilitating complications affecting the quality of life, often requiring multiple staged surgeries, and prolonged rehabilitation with physiotherapy to yield functional improvement. Wrist flexion contracture correction is the first surgery performed to reduce the deformity and improve the functional status of the hand. Releasing the wrist flexion contracture due to stretch on the contracted structures could cause a vascular compromise of the hand and skin deficit, which needs flap cover. On the other hand, removing the carpal bones reduces the length of the extremity and makes the existing skin adequate, with the wrist in the neutral position. This procedure avoids the need for a flap and avoids the stretch of blood vessels in bringing the wrist to the neutral position. A retrospective study was performed on three patients who presented to our institution, i.e., Sri Ramachandra Institute of Higher Education and Research, Chennai, India, and underwent carpectomy and wrist fusion for correction of post-traumatic wrist flexor contracture between December 2019 and July 2021, with follow-up extending to a maximum of 18 months. The three patients underwent prior surgeries at different hospitals following injury and later presented at our institution for further management and subsequently underwent surgeries and extensive rehabilitation to improve wrist and hand function. The patients underwent a staged procedure for correction of wrist contracture by soft tissue release and carpectomy, followed by wrist arthrodesis. Postoperatively, none of the patients had neurovascular complications or complications related to wound healing. Hence, carpectomy and wrist fusion are safe procedures to correct the wrist flexion contracture without complication and motivate the patient to undergo further surgeries to improve hand function.

## Introduction

The incidence of trauma has increased worldwide, especially in the younger population, as a result of increased industrialization, high-velocity road traffic accidents, and increased use of domestic and industrial motorized equipment in the current age of technological advancement. Much of the major trauma sustained requires an extended duration for treatment as well as for rehabilitation by physiotherapy to gain an adequate and satisfactory level of function. The inherent complexities associated with major trauma related to the hand and wrist, especially in developing nations, include the patient being the sole breadwinner of the family, the lack of availability of specialist surgeons, and a long course of treatment. Major trauma requires multiple staged procedures. The primary concern in the first stage is to provide a stable soft tissue cover. In injuries with skin loss, the wound has to be resurfaced with a flap cover. Most of the injuries need a secondary procedure; hence, a stable cover in the form of a flap is needed. In some major trauma, in the initial assessment, the wrist looks good, but in the later follow-up, many a time, the wrist is known to develop flexion contracture with or without a pronation contracture, not only in patients with poor compliance and lack of adequate physiotherapy. Stretching of muscle and soft tissue along with strengthening of the muscles of the hand, following a flexion contracture, show an improvement in hand function, as stretching plays a vital role in increasing the extensibility of soft tissues that have adaptively shortened due to immobilization [1].

The pathophysiology of the aforementioned complication may be due to intrinsic causes, such as ankylosis of the joint, joint locking due to mechanical obstruction, post-traumatic joint incongruity, or loose bodies. Extrinsic causes include contractures of the soft tissues, capsular thickening and fibrosis, muscle spasms, or scars [2]. Patients who sustained an injury to the wrist and hand with soft tissue injury and soft tissue loss developed post-traumatic wrist flexor contracture, thus requiring correction of the contracture to reduce the morbidity and for improvement of the functional status of the joint involved. Wrist arthrodesis with or without proximal row carpectomy (PRC) is indicated in patients with spastic hand patients, with no further recovery expected, and their deformities have not improved with conservative management, which causes hygiene problems, interfere with proper nursing care, or are causing pain due to joint subluxation or nerve compression [3]. Doing a carpectomy reduces the length of the wrist and mitigates the risk of vascularity issues, and wrist fusion reduces the step of tendon reconstruction for wrist motors. PRC is proposed as an alternative to four-corner fusion, a fusion of the capitate and perilunate, or total arthrodesis of the wrist [4]. This study was conducted to know the effectiveness of carpectomy and wrist fusion in correcting the wrist flexor contracture.

## Case presentation

Study subjects

A retrospective study was performed on patients who underwent carpectomy and wrist fusion for correction of post-traumatic wrist flexor contracture between December 2019 and July 2021 at Sri Ramachandra Institute of Higher Education and Research, Chennai, India. Three patients were recruited; the inclusion criteria included patients who underwent carpectomy for wrist flexor contracture, and the exclusion criteria included patients who were not willing to participate in the study. Pre-operative sensation assessment, muscle power grading, and range of motion were noted. The follow-up period varied from six to 18 months. All three patients were men, of which two sustained injuries due to a road traffic accident, and one of the patients sustained injury due to a workplace machine injury. All the above-mentioned patients underwent initial treatment at different hospitals but were unable to continue further treatment due to COVID-19 and presented to us for further management. The patients were planned for further subsequent surgeries for improvement of hand function.

Patient 1

**Figure 1:**
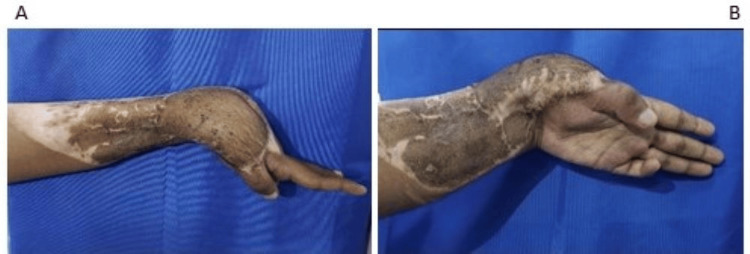
Post-traumatic flexor contracture deformity with a well-settled flap of patient 1 (A). Volar side skin grafts and ulnar deviated wrist (B).

A right-hand dominant 23-year-old male met with a road traffic accident and sustained an injury to the left wrist, for which the patient underwent multiple surgeries in the form of wound debridement with K-wire fixation, followed by superiorly based abdominal flap/split skin grafting (SSG) at another hospital. He experienced pain and deformity in the left wrist after 10 months from the time of injury. The left wrist had multiple scars, a skin graft, and a flap with a 45° fixed flexion deformity of the left wrist (Figure 1).

**Figure 2:**
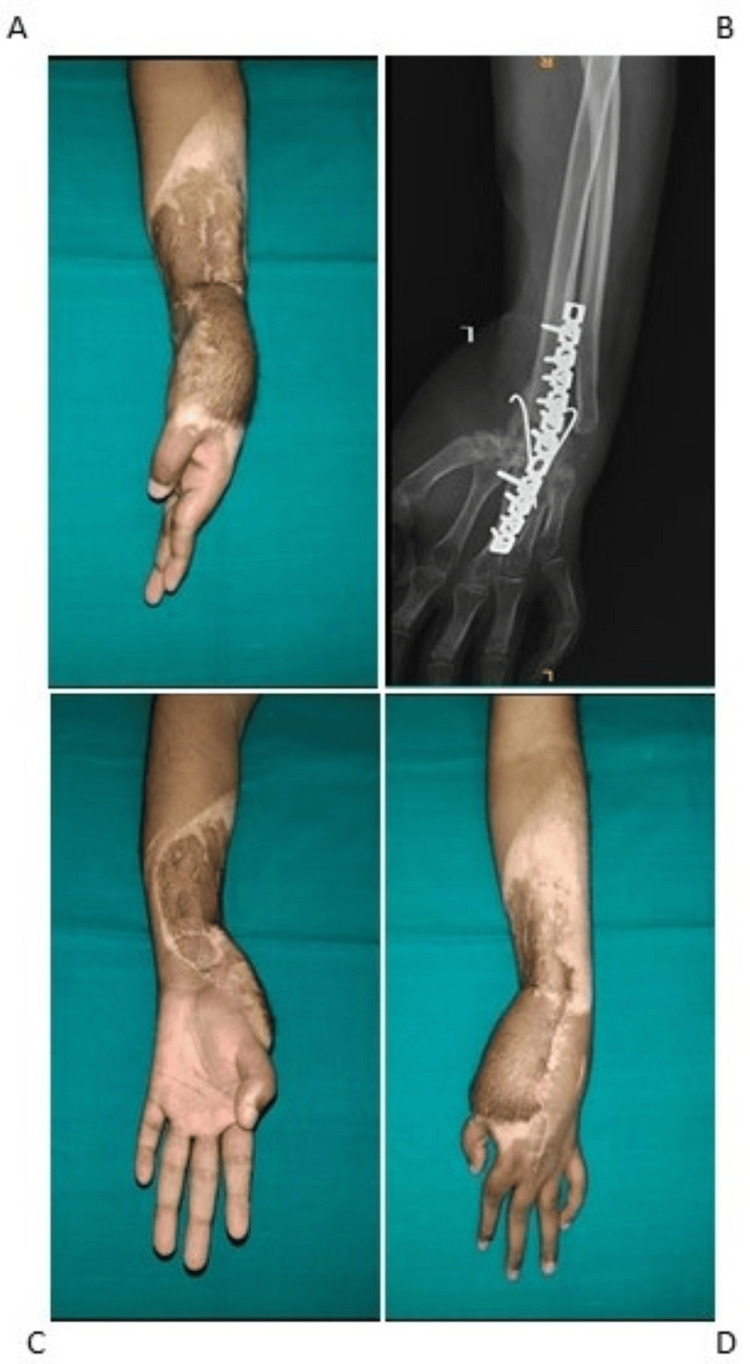
Postoperative correction of wrist flexion deformity of patient 1 (A). Postoperative X-ray of the right wrist after carpectomy and wrist arthrodesis of patient 1 (B). Reduction of deformity post-surgery on supination (C). Reduction of deformity post-surgery on pronation (D).

The main cause for the contracture was the sequelae of trauma. However, the wrist was not found to be arthritic. The patient was planned for a staged surgery. The first step in the staged approach was a correction of the wrist contracture. The patient underwent proximal and distal row corpectomy by dorsal approach, and wrist arthrodesis was performed with two K-wires of 1.8 mm size and fixed with a 12-hole reconstruction plate with the wrist fusion. The patient was planned for PRC; however, intraoperatively, the quality of bone stock was poor, and hence, all the carpal bones were removed. The patient recovered well without complications intraoperatively or postoperatively (Figure 2).

Patient 2

**Figure 3:**
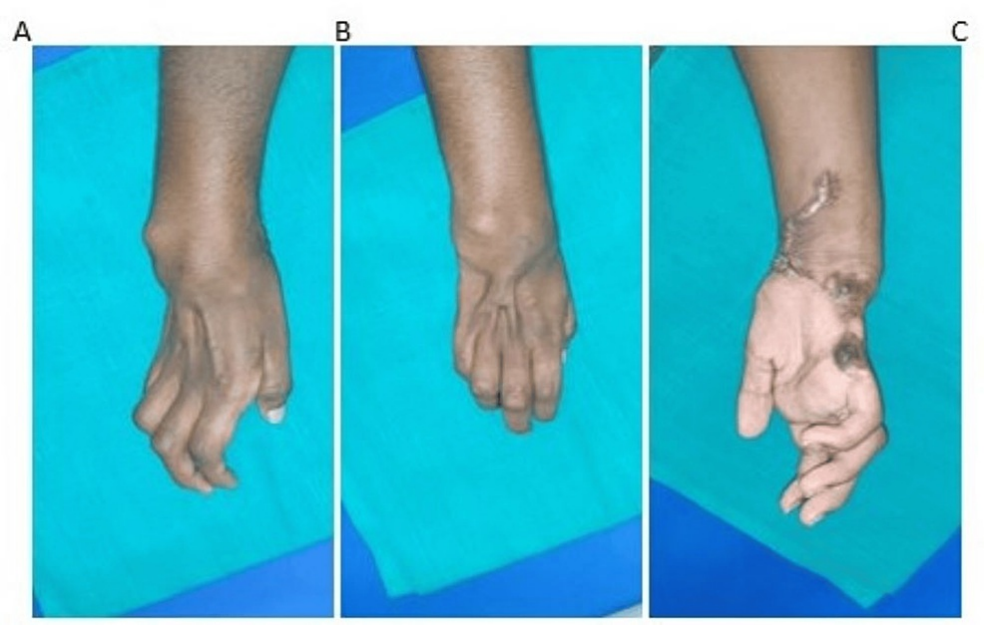
Post-traumatic flexor contracture of the wrist of patient 2 (A). The patient had severe contractures and stiff MCP joints (B). Presentation of the patient with 45° fixed flexion deformity of the wrist (C). MCP, metacarpophalangeal

A 26-year-old male presented to us with deformity of the right wrist and hand for the past two years, following post-traumatic sequelae of the right hand after sustaining an accidental machine crush injury to his right hand at his workplace about two years ago. The patient was right-hand dominant and underwent multiple procedures at another hospital and then presented to us for further management. The patient on presentation had a 45° fixed flexion deformity of the wrist (Figure 3).

**Figure 4:**
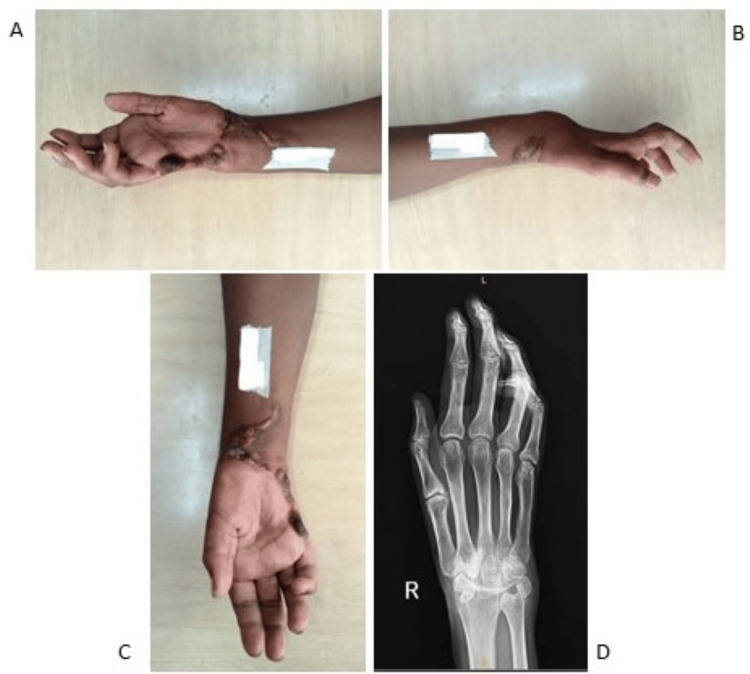
Corrected wrist deformity of patient 2 postoperatively (A). Decrease in the flexor contracture postoperatively (B). Improvement in the stiffness of MCP joint and improvement of the function of fingers postoperatively (C). Postoperative X-ray of the right wrist after carpectomy of patient 2 (D). MCP, metacarpophalangeal

The main cause for the contracture was the sequelae of trauma, and the wrist was not arthritic. The patient was planned for a staged surgery. The first step in the staged approach was a correction of the wrist contracture. The patient underwent right wrist PRC plus wrist arthrodesis. The patient also underwent an open metacarpophalangeal (MCP) joint capsulotomy for stiff MCP joints (Figure 4).

Patient 3

A right-hand dominant 39-year-old male patient presented with deformity and stiffness-flexion contracture of the right wrist, first web space contracture, and extension contracture of the fingers of three-month duration, following a circumferential degloving injury of the right hand, wrist, and forearm four months ago in a road traffic accident, for which wound debridement and SSG were performed at another hospital. On presentation, he had a circumferential SSG from the middle third-distal third junction of the forearm to MCP joint dorsum and proximal palmar crease. The MCP, proximal interphalangeal (PIP), and distal interphalangeal (DIP) joints were fixed in extension. Only a flicker of palmar flexion was present in all three joints.

Though the main cause for the contracture was because of trauma, post-traumatic wrist arthritis was present, and the patient was planned for a staged surgery. The patient had a circumferential split-thickness skin graft, and hence, the patient was planned for an Ilizarov application and distraction. The first step in the staged approach was the correction of the wrist contracture, and the patient underwent right wrist-spanning Ilizarov ring fixator application and distraction for gradual deformity correction. As it did not yield the desired results, the Ilizarov distractor was removed. Following this, a carpectomy was planned, and the patient was explained about the need for a possible flap cover in case the wound did not heal well.

**Figure 5:**
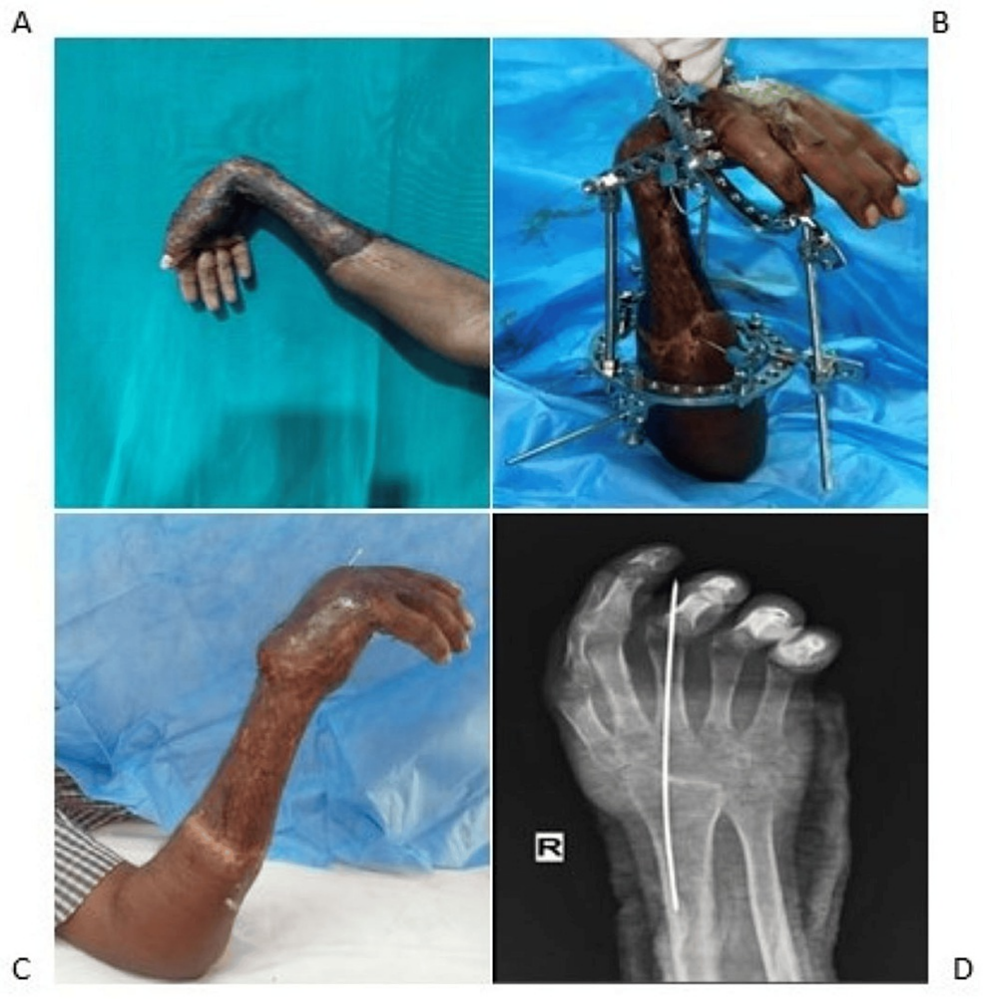
Post-traumatic wrist flexor contracture deformity of patient 3 (A). Patient 3 underwent right wrist Ilizarov ring fixator application (B). Correction of wrist flexion deformity postoperatively (C). Postoperative X-ray of the right wrist after the carpectomy (D).

The patient underwent a right wrist carpectomy by dorsal approach and arthrodesis with a single axial K-wire (Figure 5). After suture removal, a cast was applied. The patient recovered during the intraoperative and postoperative periods uneventfully.

All three patients who underwent carpectomy and fusion had regular dressings and underwent suture removal after the 10th postoperative day. They were started on the active and passive mobilization of fingers. With consistent and regular physiotherapy being provided, the grip strength and stiffness were considerably improved.

## Discussion

Wrist and hand stiffness with contracture due to post-traumatic causes is a debilitating complication affecting the patient’s day-to-day activities with a long duration and course of treatment and recovery. The flexion contracture impacts the tenodesis effect and thus the ability to grasp and release the objects. Furthermore, this large wrist flexion contracture induces extension of the MCP joints and contributes to the development of claw hand deformity [5].

In addition to the functional impairment, cosmetic blemish, especially in young patients, due to fixed deformities and contractures is a significant issue that needs to be addressed. Contractures sustained are responsible for contributing to a vast extent of problems, such as an increased disability due to decreased motor performance, mobility limitations, reduced functional range of motion, loss of function for activities of daily living (ADL), and increased pain [6].

This complication has a multifactorial pathogenesis. The importance of identifying the pathology causing the contracture necessitates that appropriate treatment cannot be understated. The various causes include (i) cicatricial skin, (ii) deep soft tissues/fascia contracture, (iii) tendon adhesions, and (iv) muscle contractures.

The pathology causing post-traumatic contracture is the shrinkage of the scar and/or adhesion of one or more injured tissues [2]. The initiation of the process of arthrofibrosis is by the excessive synovial inflammatory response due to activation with the proliferation of fibroblast cells followed by an increase in the deposition of matrix protein [7-9]. This causes inflammation of the synovium and then synovial fibrosis causing capsular thickening. This leads to contracture of the affected joint [7-10]. Preventing the development of post-traumatic flexor contracture is a better and easier modality of treatment. It is critical to remember that an acutely injured hand tends to take in an intrinsic minus position due to acute edema following injury. The injured hand gradually becomes stiff in the same position [2]. Clinical evaluations are important; however, they may not reflect the actual functional status of the patients [11]. The evaluation usually requires a holistic approach consisting of clinical examination and adequate radiographic investigation, as accuracy in diagnosis is crucial because no soft tissue release will correct a bone-related stiffness and vice versa [12].

In patients with isolated soft tissue contractures and non or minimal bone deformities, nonoperative management, such as hand therapy and splinting, is the mainstay of management [12,13]. The standard treatment for these contractures includes surgical release and skin cover. The inherent complications of the surgery include damage to the neurovascular bundles during surgery; fingertip ischemia when a chronically contracted finger is acutely straightened, outputting stretch on the neurovascular bundles; a contracted but uninjured tendon preventing the full straightening of the finger; and wound infections [14]. An increased volume of anesthesia may be necessary for the treatment of severe trauma with extensive hard scars. Moreover, soft tissue dissection may injure the reconstructed vessels and nerves [15].

For the treatment of post-traumatic arthritic wrists, PRC is an accepted motion-sparing surgical procedure [16]. The most common reason to pursue PRC is pain, but the pain is often a difficult parameter to assess. The best way to evaluate such patients is by using analog pain evaluation instruments and objective findings [17]. PRC is a useful procedure for various conditions such as degenerative joint arthritis regardless of cause, including idiopathic, rheumatoid arthritis, and osteoarthritis [17]. PRC results in significant pain relief, satisfactory strength, and high patient satisfaction [16]. In patients with irreversible joint destruction, total wrist arthrodesis provides stability, strength, and pain relief [18]. Intercarpal arthrodesis is a bone reconstructive technique that helps to retain motion at the wrist [17].

One of the techniques that have been proven highly predictable and successful for arthrodesis of the wrist involves combining rigid internal fixation with concomitant removal of the proximal carpal row bones, which are then morselized and used for bone grafting. No distant bone graft site is required, and rigid internal fixation that allows virtually immediate rehabilitation is the major advantage of employing this technique [19]. PRC performed concomitantly with total wrist arthrodesis would be expected to improve healing rates, as a single joint space must undergo fusion for the procedure to be successful [18]. Patient-reported outcome measures are reflections of the functional status of patients [11]. The assessment and evaluation for the need of the procedure are patient-specific, and the risk-benefit ratio is assessed over the potential known complications for spanning wrist arthrodesis, including flexor or extensor tendinopathy, peripheral neuropathy, radiocarpal non-union, carpometacarpal non-union, peri-implant failure, and hardware breakage. Carpectomy thus reduces the shortening of the extensor and flexor tendon with wrist contracture. The primary wrist arthrodesis goals were for stability and hygiene, with functional increases and avoidance of the weakening of flexor made secondary. The use of compression plates allowed the patient to be free from splints or casts in the early postoperative period to allow ease of nursing care to improve hygiene [3]. We were at a disadvantage because all three patients returned to their primary care provider once COVID-19 restrictions were lifted. Hence, adequate and serial assessment of functional outcomes and improvement were limited. In addition to this, it has to be emphasized that despite the improvement of the function of the wrist and hand in terms of improving grip strength, intrinsic muscle function of the hand, and functional position of the wrist and hand along with improvement of cosmesis of the wrist and hand following carpectomy and arthrodesis supplemented with adequate physiotherapy, the complete functional outcome cannot be commented upon, as further staged procedures are required to gain the maximum function possible. The treatment of wrist joint contracture with an external fixator requires more time and cost, as well as regular maintenance of the pin tracts [15]. The limitations of the study are a small number of patients and a lack of adequate postoperative functional assessment.

## Conclusions

In the postoperative period, none of the patients experienced neurovascular complications or complications related to wound healing. The patients underwent sensation assessment, muscle power grading, and range of motion assessment by the surgeon and hand therapist.

Removal of the proximal row reduces the stretch of the blood vessels. Reduction of bone length compensates for the skin deficit; hence, there is no need for a flap cover to address the skin deficiency. Though our series is a small one, we would recommend a wrist-spanning external fixator for six to 12 weeks in major circumferential injuries involving the hand, wrist, and forearm. In addition to the surgical intervention, hand therapy plays a vital role in improving the grip strength, intrinsic muscle function, and stretching of the wrist and hand to improve the functional outcome of the patients.

Thus, we conclude that PRC with wrist fusion is a safe procedure with fewer complications, which include a problem with wound healing, extended and prolonged duration of treatment with rehabilitation, and the need for further surgeries, which were explained to the patient. However, the correction of wrist flexion contracture deformity improved the appearance of the hand and wrist deformity, which plays a major role psychologically and motivates the patients to undergo further surgeries to improve the hand function. In addition to this, the patient was able to maintain hand hygiene of the affected hand after the procedure as well, and it aided in better personal hygiene of the patient.
